# Relationship Between Patient and Informant Assessment of Personality and Cognitive Status

**DOI:** 10.1093/geroni/igaa057.834

**Published:** 2020-12-16

**Authors:** Magdalena Tolea, James Galvin

**Affiliations:** 1 University of Miami Miller School of Medicine, Boca Raton, Florida, United States; 2 University of Miami Miller School of Medicine, Palm Beach Gardens, Florida, United States

## Abstract

Personality has been linked to risk of dementia. Most studies ask individuals to rate their own personality traits or for a knowledgeable informant to perform the rating; few collect data from both. When informants are asked to give an estimate of the patient’s lifelong personality traits, they often describe personality before disease onset. When asked to self-rate, patients may instead assess their personality as they see themselves, providing a personality-state measure. The goal of this study was to assess agreement between two independent measures of personality and evaluate whether stage of cognitive impairment and characteristics of patients or caregivers impact concordance. In 79 consecutive patient-caregiver dyads presenting to our center (mean age:76.8±8.4; 44.1% female; 6% cognitively normal, 41% MCI; and 53% dementia) with in-depth psychosocial and neuropsychological evaluations, we found informants rated patients lower on openness (O) (ICC=0.434; 95%CI: 0.235-0.598) and agreeableness (A) (ICC=0.491; 95%CI: 0.302-0.643) and higher on extraversion (O) (ICC=0.396; 95%CI: 0.191-0.568) and neuroticism (N) (ICC=0.444; 95%CI: 0.247-0.607). Greater discordance was observed in established dementia (ICCE=0.497; 95%CI: 0.222-0.700; ICCA=0.337; 95%CI:0.031-0.586; ICCN=0.422; 95%CI: 0.191-0.683), compared with MCI (ICCO=0.568; 95%CI: 0.282-0.762). We explored the effect of patient and caregiver mood and caregiver burden on personality ratings. Although personality is typically described as a trait, we present evidence that in the eyes of patients, personality ratings may represent a state that changes across the spectrum of cognitive impairment. Understanding how patients and caregivers differentially perceive personality may assist in developing novel psychotherapeutic interventions and approaches dealing with behavioral manifestations of dementia.

